# Characterization and Phylogenetic Analysis of the Chloroplast Genomes of *Stephania japonica* var. *timoriensis* and *Stephania japonica* var. *discolor*

**DOI:** 10.3390/genes15070877

**Published:** 2024-07-03

**Authors:** Li-Li Wu, Ying-Min Geng, Lan-Ping Zheng

**Affiliations:** College of Chinese Materia Medica, Yunnan University of Chinese Medicine, Kunming 650500, China; wll0328211@163.com (L.-L.W.); gengym1998@126.com (Y.-M.G.)

**Keywords:** *Stephania*, chloroplast genome, high-throughput sequencing, phylogeny

## Abstract

This study sequenced the complete chloroplast genomes of *Stephania japonica* var. *timoriensis* and *Stephania japonica* var. *discolor* using the Illumina NovaSeq and PacBio RSII platforms. Following sequencing, the genomes were assembled, annotated, comparatively analyzed, and used to construct a phylogenetic tree to explore their phylogenetic positions. Results indicated that the chloroplast genomes of *S. japonica* var. *timoriensis* and *S. japonica* var. *discolor* both displayed a typical double-stranded circular tetrameric structure, measuring 157,609 and 157,748 bp in length, respectively. Each genome contained 130 annotated genes, with similar total GC content and relative codon usage patterns, showing a distinct preference for A/U at the third codon position. Simple sequence repeat analysis identified 207 and 211 repeats in *S. japonica* var. *timoriensis* and *S. japonica* var. *discolor*, respectively, primarily the A/T type. Boundary condition analysis indicated no significant expansion or contraction in the inverted repeat regions with consistent gene types and locations across both varieties. Nucleotide polymorphism analysis highlighted greater variation in the intergenic regions than in the coding sequences of *Stephania* chloroplast genomes. Phylogenetic analyses demonstrated that the species *Stephania* clustered into a distinct, well-supported clade. Notably, *Stephania japonica*, along with *S. japonica* var. *discolor* and *S. japonica* var. *timoriensis*, established a monophyletic lineage. Within this lineage, *S. japonica* and *S. japonica* var. *discolor* were closely related, with *S. japonica* var. *timoriensis* serving as their sister taxon.

## 1. Introduction

The genus *Stephania*, mainly consisting of herbaceous or woody vines, belongs to the family Menispermaceae, renowned for its medicinal properties [1]. This genus has long been used medicinally in China, first recorded in the *Supplement to Materia Medica* from the Dynasty Tang [2]. Currently, there are approximately 60 known species of *Stephania* worldwide, including 39 species and varieties in China alone [1]. This genus is primarily distributed in the Yangtze River basin and Southern China, particularly in the Guangxi and Yunnan province, and is commonly used as traditional Chinese medicine in its local area [3,4]. The challenge of the *Stephania* genus arises from the morphological similarities and overlapping habitats of its species, complicating accurate identification and increasing the risk of unintentional mixing during medicinal preparation [5,6,7,8]. Consequently, collecting genetic data for the genus is essential to addressing these issues.

In China, the genus *Stephania* is classified into three subgenera, *Botryodiscia*, *Stephania*, and *Tuberiphania* [9]. Notably, *Stephania tetrandra* S. Moore is recognized as a distinct subgenus due to its unique biological characteristics and chemical properties, as documented in Part I of the Chinese Pharmacopoeia [10,11]. *Tuberiphania* includes the largest number of species within the genus [12,13]. To date, research efforts have predominantly focused on the subgenera *Botryodiscia* and *Tuberiphania*, including their morphology, chemical composition, and pharmacological effects [14,15,16,17,18,19]. In contrast, the subgenus *Stephania* has received less attention, with four species and varieties classified under Sect. *Stephania*, including *S. japonica* var. *timoriensis* and *S. japonica* var. *discolor*, varieties of *S. japonica* [1]. While the chloroplast genome of *S. japonica* has been characterized [20], the complete chloroplast genome of *S. japonica* var. *timoriensis* and *S. japonica* var. *discolor* have yet to be reported. Given this knowledge gap, it is crucial to augment the chloroplast genome data for these variants to better understand the interspecific relationships within the *Stephania* genus.

Chloroplasts, the primary sites for photosynthesis, possess their own genetic system, commonly referred to as chloroplast genomes [21]. Given the recent advancements in sequencing technology, the chloroplast genome has emerged as a vital tool for delineating species differences and addressing phylogenetic issues, especially in medicinal plants, becoming increasing prevalent in both plant taxonomy and medicinal plant research [22,23,24,25].

Chloroplast genome sequencing has evolved to incorporate third-generation technologies, which, while slightly more costly, offer greater accuracy and completeness compared to the second-generation technologies known for their shorter read lengths and reduced costs [26]. Currently, a hybrid approach combining both second-generation and third-generation sequencing technologies is often employed in chloroplast genome research [27]. In the present study, the chloroplast genomes of *S. japonica* var. *timoriensis* and *S. japonica* var. *discolor* were sequenced using a combination of second- and third-generation sequencing technologies. Furthermore, the genome characteristics of *Stephania* were compared, and phylogenetic analyses were conducted. This research is expected to provide valuable reference data for genetic diversity and phylogenetic research within the genus *Stephania*.

## 2. Materials and Methods

### 2.1. Sample Collection, DNA Extraction, and Sequencing

Specimens of *S. japonica* var. *timoriensis* and *S. japonica* var. *discolor* were collected from the Gaoligong Mountain in Yunnan, China (25°5′9″ N, 98°48′46″ E; 25°18′2″ N, 98°48′07″ E), while additional samples were downloaded from GenBank (Table 1). Fresh leaves with good growth status were collected, frozen with liquid nitrogen, and stored at −80 °C until further use. Genomic DNA was extracted using a plant DNA extraction kit (Beijing TransGen Biotech Co., Ltd., Beijing, China). For sequencing, a library with 300 bp–500 bp insertion fragments and 150 bp read lengths was prepared using the Illumina NovaSeq platform. A separate library with a 10 kb insertion fragment was created using the PacBio RS II platform. Trimmomatic v0.39 [28] was used to trim the Illumina raw data, and Pacbio data were filtered to obtain clean reads.

### 2.2. CpDNA Assembly and Annotation

The Illumina datasets were initially assembled using GetOrganelle v1.7.5, followed by the assembly of the second- and third-generation data using SPAdes v3.14.1 [29,30]. The chloroplast genomes were annotated using GeSeq (https://chlorobox.mpimp-golm.mpg.de/geseq.html (accessed on 4 December 2023)) and verified manually [31]. The chloroplast genomes of *S. japonica* var. *timoriensis* and *S. japonica* var. *discolor* were submitted to the National Center for Biotechnology Information (NCBI) database under the accession numbers PP175340 and PP175339. Visualization of the chloroplast genome maps was accomplished using the OGDRAW online tool (https://chlorobox.mpimp-golm.mpg.de/OGDraw.html (accessed on 3 February 2024)).

### 2.3. Codon Preference Analysis and Repeat Sequence Analysis

CodonW v1.4.2 was used to calculate the relative synonymous codon usage (RSCU), total GC content, and the GC content of each codon within the chloroplast genomes [32]. This analysis followed an initial screening of the chloroplast genome protein-coding sequences based on specific criteria as follows: (1) start codon of ATG and stop codon of TAA, TAG, or TGA; (2) sequence length of at least 300 bp; and (3) deletion of any repetitive sequences [33]. Analysis of simple sequence repeats (SSRs) within the chloroplast genome sequences of the two species was conducted using MISA [34], with parameters of repeat units set to ≥10 for mononucleotides, ≥5 for dinucleotides, ≥4 for trinucleotides, and ≥3 for tetranucleotides, pentanucleotides, and hexanucleotides. The minimum distance between two SSRs was set to 100 bp.

### 2.4. Comparative Genomics Analysis of the Chloroplast Genome

Boundary regions of the chloroplast genomes from eight species of *Stephania*, including *S. japonica* var. *timoriensis* and *S. japonica* var. *discolor*, were examined using IRscope [35], assessing the contraction and expansion of these regions by comparing boundary genes. Comparative genomic analysis across these species was conducted using mVISTA with the Shuffle–LAGAN model [36]. Nucleotide polymorphisms (Pi) across common genes and intergenic regions of the chloroplast genomes were calculated using DnaSP v.6.12.03 [37].

### 2.5. Phylogenetic Analysis

To investigate the phylogenetic positions and affinities of *S. japonica* var. *timoriensis* and *S. japonica* var. *discolor*, chloroplast genome sequences from six species of *Stephania*, five species of the family Menispermaceae, two species of the family Lardizabalaceae, two species of the family Ranunculaceae, and five species of the family Berberidaceae were downloaded from GenBank for phylogenetic tree construction. Sequence alignment was performed using MAFFT, and the phylogenetic tree was constructed using IQ-TREE, based on the maximum likelihood (ML) method with the following parameters: −m MFP −bb 1000 −alrt 1000 −nt 4 [38,39].

## 3. Results

### 3.1. Analysis of Characteristics of Chloroplast Genomes

**Figure 1:**
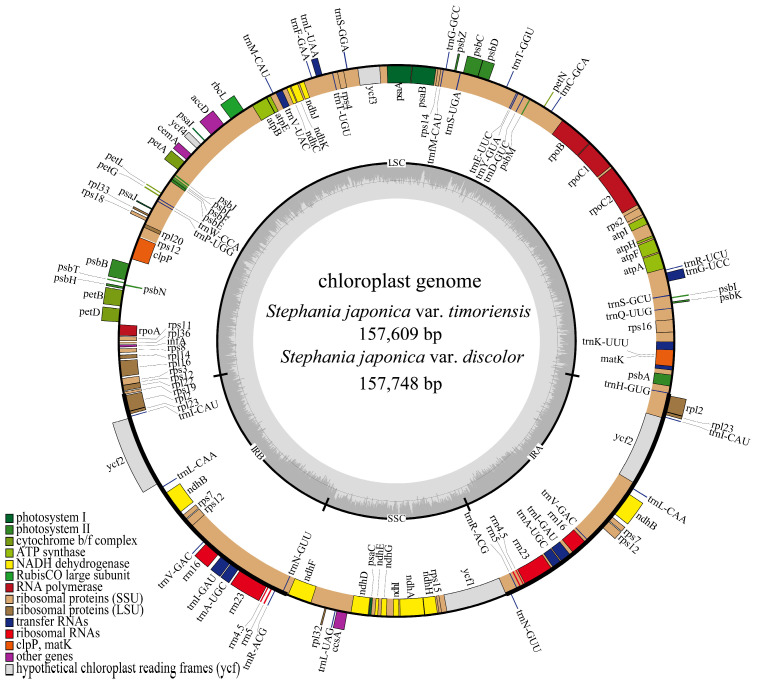
Chloroplast genomes of *S. japonica* var. *timoriensis* and *S. japonica* var. *discolor*.

A total of 8449 Mb and 6862 Mb of Illumina raw sequencing data, as well as 2245 Mb and 630 Mb PacBio raw data, were obtained for *S. japonica* var. *timoriensis* and *S. japonica* var. *discolor*, respectively. The assembled chloroplast genomes of *S. japonica* var. *timoriensis* and *S. japonica* var. *discolor* were 157,609 bp and 157,748 bp in length, respectively (Table 2), composed of double-stranded circular DNA with typical tetrameric structures, including one large single copy (LSC), one small single copy (SSC) and two inverted repeats (IR) (Figure 1). The LSC regions of *S. japonica* var. *timoriensis* and *S. japonica* var. *discolor* were 88,477 bp and 88,581 bp, respectively, while the SSC regions were 20,346 bp and 20,365 bp and the IR regions were 24,393 bp and 24,401 bp (Table 2). Both genomes exhibited a total GC content of 38.25% and 38.26% (Table 2), with highest GC content observed in the IR region (43.72% and 43.73%), followed by the LSC (36.46% and 36.46%) and SSC regions (32.94% and 32.97%).

Annotation of the chloroplast genomes of *S. japonica* var. *timoriensis* and *S. japonica* var. *discolor* yielded 130 genes, including 85 protein-coding genes (PCGs), 37 transfer RNA genes (tRNAs), and 8 ribosomal RNA genes (rRNAs) (Table 3). Among them, six PCGs (*ndhB*, *rpl2*, *rpl23*, *rps7*, *rps12*, and *ycf2*), seven tRNAs (*trnA-UGC*, *trnI-CAU*, *trnI-GAU*, *trnL-CAA*, *trnN-GUU*, *trnR-ACG*, and *trnV-GAC*), and four rRNAs (*rrn4.5S*, *rrn5S*, *rrn16S*, and *rrn23S*) were located in the IR region, each with two copies. All other genes were present as single copies. The genes in the chloroplast genome were categorized into four functional groups. The first group consisted of 45 genes related to photosynthesis, including 5 photosystem I genes, 15 photosystem II genes, 6 cytochrome b/f complex genes, 12 NADH dehydrogenase genes, 6 ATP synthase genes, and 1 Rubisco subunit gene. The second group consisted of 74 genes related to self-replication, including 11 ribosomal large subunit genes, 14 ribosomal small subunit genes, 4 RNA polymerase genes, in addition to tRNA genes and rRNA genes. The third group consisted of six genes for various proteins, while the fourth group consisted of five genes with unknown functions.

### 3.2. Codon Usage Bias

**Figure 2:**
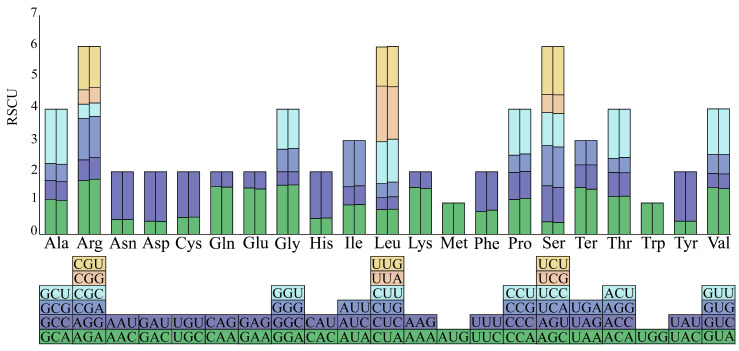
Relative synonymous codon usage of chloroplast genomes of *S. japonica* var. *timoriensis* and *S. japonica* var. *discolor*. Histogram of each amino acid from left to right is *S. japonica* var. *timoriensis* and *S. japonica* var. *discolor*.

A total of 50 protein-coding sequences from the *S. japonica* var. *timoriensis* and *S. japonica* var. *discolor* chloroplast genomes were analyzed for codon usage preferences (Figure 2). The codon usage patterns of chloroplast genome across both varieties were largely similar, exhibiting only minor variations. Codon analysis revealed that each genome contained 64 codons, with total usage frequencies of 20,002 and 22,022, respectively. Excluding the 3 termination codons, the remaining 61 codons encoded 20 amino acids. Notably, codons for leucine (Leu) were the most abundant in the two chloroplast genomes, accounting for 10.15% (2031) and 10.23% (2252) of total codons, respectively, while the codons for cysteine (Cys) were the least abundant, accounting for 1.16% (233) and 1.17% (258), respectively. Most amino acids were encoded by 2–6 synonymous codons, except for methionine (Met) and tryptophan (Trp), which were uniquely encoded by the single codons AUG and UGG, respectively, both with an RSCU value of one, indicating no preference in usage. When RSCU is larger than one, it indicates a stronger preference for using this codon to encode the same amino acid as compared to other synonymous codons. When RSCU is less than one, it is lower than that of other synonymous codons in usage. In the chloroplast genomes of these two varieties, except two codons with an RSCU of one, there were 62 remaining codons, including 31 codons with an RSCU greater than one, suggesting a preference for these codons, and 31 codons with an RSCU less than one, indicating lesser usage. Among these, 29 preferred codons ending in A or U, with only 3 not preferring codons ending in A or U, suggesting that high-frequency codons tend to end in A/U and low-frequency codons in G/C. The highest RSCU values in the chloroplast genomes of *S. japonica* var. *timoriensis* and *S. japonica* var. *discolor* were for UUA encoding Leu and AGA encoding arginine (Arg), respectively. Total GC content in the codons encoding amino acids was 38.7% for both varieties, with the third codon showing GC content of 27.9% and 28.6%, respectively. This pattern indicates a bias towards A/U-rich codons, especially at the third codon position in both varieties.

### 3.3. Repeat Sequences and SSR Analysis

A total of 207 and 211 SSRs were identified in the chloroplast genomes of *S. japonica* var. *timoriensis* and *S. japonica* var. *discolor*, respectively. These SSRs comprised five types, including mononucleotide, dinucleotide, trinucleotide, tetranucleotide, and pentanucleotide repeat sequences, with no hexanucleotide repeats detected. As shown in Table 4, the SSRs were primarily concentrated in the LSC region, followed by the SSC region, with the fewest found in the IR region. Specifically, the LSC region contained 142 and 143 SSRs, the SSC region contained 45 and 48 SSRs, and the IR region contained 20 and 20 SSRs in *S. japonica* var. *timoriensis* and *S. japonica* var. *discolor*, respectively. Mononucleotide repeats were the most common, accounting for 81.2% (168) and 83.4% (176) of all SSRs, mostly the A/T type, followed by dinucleotide repeats (18), mainly the AT/AT type. The remaining sequences accounted for a relatively small proportion, including seven and six trinucleotide repeats, nine and seven tetranucleotide repeats, and five and four pentanucleotide repeats in *S. japonica* var. *timoriensis* and *S. japonica* var. *discolor*, respectively.

### 3.4. Comparative Genome Analysis

**Figure 3:**
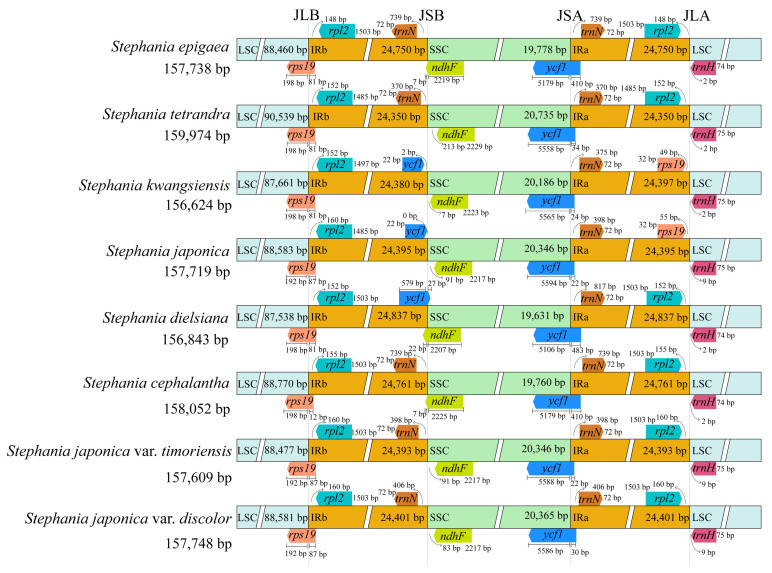
Comparison of LSC, IRs, and SSC border regions of chloroplast genomes of *Stephania*.

The chloroplast genomes of the eight *Stephania* species exhibited minimal differences in total length and within their four zones, indicating a high level of conservation (Figure 3). However, the genes at the boundaries were not completely identical, displaying some expansion and contraction. The boundaries between two adjacent single copy regions and the IR region were designated as JLB, JSB, JSA, and JLA, respectively. The JLB boundaries of all eight species consistently fell within the *rps19* gene, to the left of the *rpl2* gene. The JSA boundaries consistently occurred within the *ycf1* gene, to the left of the *trnN* gene. There were slight differences in the degree of expansion between these eight species. The genes at the JSB boundary of *S. japonica* var. *timoriensis*, *S. japonica* var. *discolor*, *Stephania epigaea*, *S. tetrandra*, and *Stephania cephalantha* were *trnN* and *ndhF*, while the genes at the JSB boundary of the remaining species were *ycf1* and *ndhF*. At the JLA boundary, *Stephania kwangsiensis* and *S. japonica* had the same flanking genes, *rps19* and *trnH*, whereas the other six species, including *S. japonica* var. *timoriensis* and *S. japonica* var. *discolor*, contained the flanking genes *rpl2* (in the IRa region) and *trnH* (in the LSC region), with a distance of 148–160 bp from the end of *rpl2* and the JLA boundary. In this study, the boundary gene types of *S. japonica* var. *timoriensis* and *S. japonica* var. *discolor* were completely identical, and the distances between the flanking genes and boundaries were similar, with no significant contraction or expansion observed.

**Figure 4:**
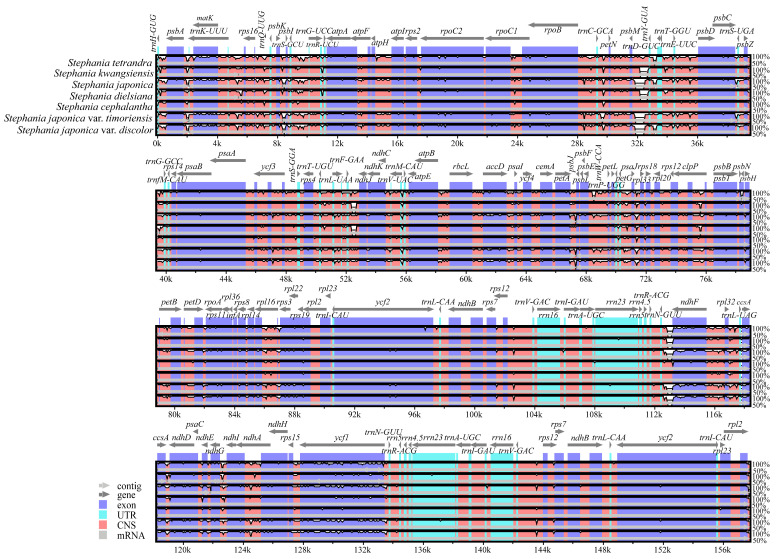
Comparative analysis of chloroplast genomes of *Stephania*.

To assess the extent of chloroplast genome variation within the genus, comprehensive sequence alignment was performed on the chloroplast genomes of seven other *Stephania* species, using *S. epigaea* as a reference. Overall, the *Stephania* chloroplast genomes exhibited high similarity and conservation. The LSC and SSC regions showed greater variability compared to the IR region, with non-coding regions displaying significantly higher variation than coding regions. The tRNA and rRNA sequences within the IR region were highly conserved (Figure 4).

**Figure 5:**
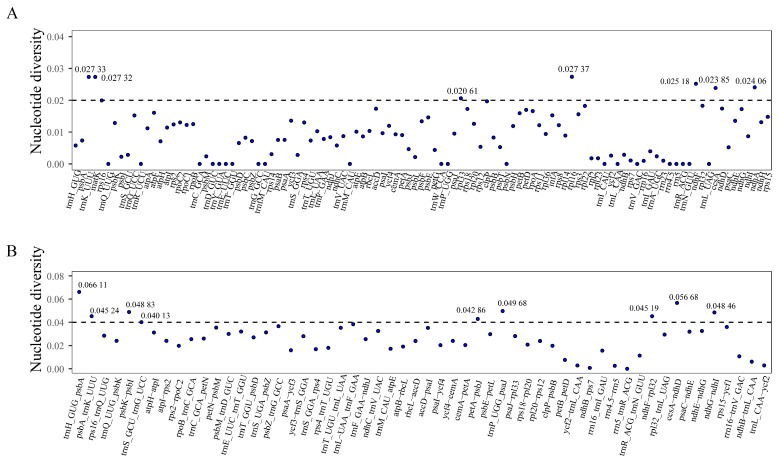
Nucleic acid polymorphism analysis of chloroplast genomes of *Stephania*. (**A**) Nucleic acid polymorphism analysis of common genes. (**B**) Nucleic acid polymorphism analysis of common intergenic regions.

A total of 107 common gene sequences and 56 common spacer sequences were extracted from the chloroplast genomes of *Stephania*. The Pi values for the common spacer sequences were generally higher than those for the common gene sequences. Among the shared gene sequences, the Pi values for *rpl33*, *ccsA*, *ndhA*, *ndhF*, *matK*, *trnK-UUU*, and *rpl16* exceeded 0.020. Among the shared spacer sequences, the Pi values of *trnS-GCU-trnG-UCC*, *petA-psbJ*, *ndhF-rpl32*, *psbA-rnK-UUUU*, *ndhG-ndhI*, *psbK-psbI*, *trnP-UGG-psaJ*, *ccsA-ndhD*, and *trnH-GUG-psbA* exceeded 0.040 (Figure 5). These highly mutated regions could serve as potential molecular markers for species identification within the genus *Stephania*.

### 3.5. Phylogenetic Analysis

**Figure 6:**
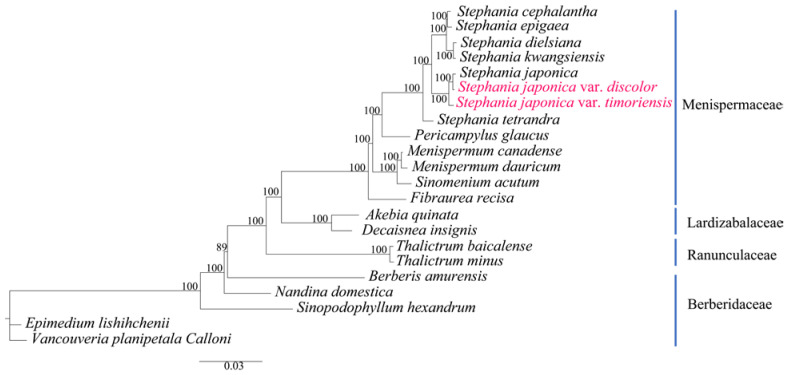
Maximum likelihood (ML) phylogenetic tree of analysis based on chloroplast genome sequences of 22 species. The species highlighted in pink font color are newly sequenced in this study.

Phylogenetic analysis indicated that the support rate for all lineages was greater than 85%, confirming the reliability of the results. All species within the family Menispermaceae formed a monophyletic branch. Within this framework, the eight *Stephania* species clustered into one lineage with 100% support. The genus was further divided into three lineages. *S. tetrandra* formed an independent lineage, while *S. japonica*, *S. japonica* var. *discolor* and *S. japonica* var. *timoriensis* formed a monophyletic lineage with 100% support, and *S. japonica* var. *discolor* and *S. japonica* formed a lineage as sister taxon to *S. japonica* var. *timoriensis*. Furthermore, *S. cephalantha* and *S. epigaea* formed a distinct lineage which was the closest relative to the lineage formed by *Stephania dielsiana* and *S. kwangsiensis*. These four species collectively constituted the third lineage within the genus *Stephania* (Figure 6).

## 4. Discussion

In this study, we assembled the chloroplast genomes of *S. japonica* var. *timoriensis* and *S. japonica* var. *discolor*. Consistent with other angiosperms, the chloroplast genomes of these two species exhibited a typical four-part structure, including the LSC, SSC, IRa, and IRb regions. The chloroplast genomes of the two species showed remarkable similarity in length, total GC content, and GC content of each partition. Additionally, the number, type, and arrangement of genes were highly consistent with previously reported *Stephania* species [40,41]. The *Stephania* chloroplast genomes exhibited a degree of conservation; however, they differed from other genera within Menispermaceae, such as *Menispermum*, *Sinomenium*, and *Fibraurea*, which had longer chloroplast genomes (exceeding 160,000 bp) and lower total GC content [42,43,44].

Our analysis also showed that the boundary wing gene types in the chloroplast genomes of the two varieties were identical. However, the genotypes at JSB and JLA differed among the eight *Stephania* species, particularly at the location of the *ndhF* gene, which occurred in either the SSC region or at the IRb/SSC junction. These differences are likely due to varying degrees of IR region constriction or expansion, which are primary factors contributing to variations in chloroplast genomes [45]. The preference for codon usage is important for understanding species evolution and gene expression. Our analysis revealed a high degree of similarity in codon usage between the two varieties, with both showing a preference for A/U, likely due to the high content of the A and T bases, resulting in a bias towards A or T ending codons. The RSCU values indicated that the codons for Leu and Arg had the highest frequency of occurrence. These codon preference results are consistent with previously reported findings for *S. tetrandra* [40].

The identification of SSRs is crucial for investigating and identifying genetic diversity at the molecular level. In this study, 207 and 211 SSRs were identified in the chloroplast genomes of *S. japonica* var. *timoriensis* and *S. japonica* var. *discolor*, respectively. These SSRs were diverse in type and primarily distributed in the LSC region, consistent with other angiosperms [23,24,46]. These SSR loci are promising candidates for future molecular identification of *Stephania* species. By examining the sequence variations in the chloroplast genome of the genus *Stephania*, we identified seven common gene sequences with Pi values greater than 0.020 and nine common inter-region sequences with Pi values of greater than 0.040 in the highly variable region. In previous studies, Wang et al. identified one of the six candidate DNA barcodes identified in our results (*matk*), while Dong et al. identified two of the five high Pi value regions consistent with our findings (*trnH-psbA*, *ndhA*) [40,47]. In addition to these three reported molecular markers, we screened 13 mutation hotspots, including *rpl33*, *ccsA*, *ndhF*, *trnK*, *rpl16*, *trnS-trnG*, *petA-psbJ*, *ndhF-rpl32*, *psbA-trnK*, *ndhG*-*ndhI*, *psbK-psbI*, *trnP-psaJ*, and *ccsA-ndhD*. These highly variable sequences could serve as a potential tool for identifying different species within the genus *Stephania*.

The phylogenetic tree constructed from the chloroplast genomes of *S. japonica* var. *timoriensis*, *S. japonica* var. *discolor*, and 20 related species demonstrated that all *Stephania* species formed a monophyletic group, clearly divided into three lineages, corresponding to the subgenera *Botryodiscia*, *Stephania*, and *Tuberiphania. Stephania* and *Tuberiphania* diverged from the same node, indicating a close relationship. Based on phylogenetic analysis of the nuclear transcribed spacer (*ITS*) and chloroplast transcribed spacer (*trnL-F*) sequences, Xie et al. also divided *Stephania* into three major lineages [48], consistent with our findings, but did not fully resolve the interrelationships among the three lineages due to low support values [48]. In our study, *S. japonica*, *S. japonica* var. *discolor*, and *S. japonica* var. *timoriensis* were clustered within the subgenus *Stephania*. Specifically, *S. japonica* and *S. japonica* var. *discolor* formed a lineage that was the sister taxon to *S. japonica* var. *timoriensis*. These findings align with the phylogenetic tree constructed based on *ITS*+*psbA-trnH* in Wang et al. [47]. However, our internal branching results of the subgenus *Tuberiphania* differ slightly from those of Wang et al. [47], particularly regarding the relationship between *S. epigaea* and *S. cephalantha*. These preliminary phylogenetic results indicate that the taxonomic status of the two varieties needs to be further examined. Further research including more species is necessary to determine the phylogenetic positions within *Stephania*. Additionally, expanding the chloroplast genome data for other species in this genus is essential.

## 5. Conclusions

In this study, the chloroplast genomes of *S. japonica* var. *timoriensis* and *S. japonica* var. *discolor* were obtained using second- and third-generation sequencing techniques. This study enriches the chloroplast genome data within the genus *Stephania* and provides valuable molecular data for the precise identification of similar species, as well as for studying genetic diversity in the genus *Stephania*.

## Figures and Tables

**Table 1:** Samples used in this study.

No.	Species	From	GenBank Accession No.
1	*Stephania japonica* var. *timoriensis*	This study	PP175340
2	*Stephania japonica* var. *discolor*	This study	PP175339
3	*Stephania cephalantha*	GenBank	NC_067079
4	*Stephania dielsiana*	GenBank	NC_054337
5	*Stephania epigaea*	GenBank	NC_058988
6	*Stephania japonica*	GenBank	NC_029432
7	*Stephania kwangsiensis*	GenBank	NC_048524
8	*Stephania tetrandra*	GenBank	NC_050924
9	*Pericampylus glaucus*	GenBank	NC_046846
10	*Menispermum canadense*	GenBank	NC_048451
11	*Menispermum dauricum*	GenBank	NC_042371
12	*Sinomenium acutum*	GenBank	MT040976
13	*Fibraurea recisa*	GenBank	NC_060536
14	*Akebia quinata*	GenBank	KX611091
15	*Decaisnea insignis*	GenBank	KY200671
16	*Thalictrum baicalense*	GenBank	MW133265
17	*Thalictrum minus*	GenBank	NC_041544
18	*Berberis amurensis*	GenBank	KM057374
19	*Nandina domestica*	GenBank	DQ923117
20	*Sinopodophyllum hexandrum*	GenBank	KR779994
21	*Epimedium lishihchenii*	GenBank	KU522472
22	*Vancouveria planipetala*	GenBank	MH337373

**Table 2:** Composition and characteristics of chloroplast genomes of *S. japonica* var. *timoriensis* and *S. japonica* var. *discolor*.

Species	Genome		LSC		IR		SSC	
	Length/bp	GC/%	Length/bp	GC/%	Length/bp	GC/%	Length/bp	GC/%
*S. japonica* var. *timoriensis*	157,609	38.25	88,477	36.46	24,393	43.72	20,346	32.94
*S. japonica* var. *discolor*	157,748	38.26	88,581	36.46	24,401	43.73	20,365	32.97

**Table 3:** Functional annotation and classification of chloroplast genomes of *S. japonica* var. *timoriensis* and *S. japonica* var. *discolor*.

Gene Function	Gene Group	Gene Name	Number
Gene for photosynthesis	Photosystem I	*psaA*, *psaB*, *psaC*, *psaI*, *psaJ*	5
	Photosystem II	*psbA*, *psbB*, *psbC*, *psbD*, *psbE*, *psbF*, *psbH*, *psbI*, *psbJ*, *psbK*, *psbL*, *psbM*, *psbN*, *psbT*, *psbZ*	15
	Cytochrome b/f complex	*petA*, *petB*, *petD*, *petG*, *petL*, *petN*	6
	NADH dehydrogenase	*ndhA*, *ndhB* ^a^, *ndhC*, *ndhD*, *ndhE*, *ndhF*, *ndhG*, *ndhH*, *ndhI*, *ndhJ*, *ndhK*	12
	Subunits of ATP synthase	*atpA*, *atpB*, *atpE*, *atpF*, *atpH*, *atpI*	6
	Rubisco large subunits	*rbcL*	1
Self-replication	Lange subunits of ribosome	*rpl2* ^a^, *rpl14*, *rpl16*, *rpl20*, *rpl22*, *rpl23* ^a^, *rpl32*, *rpl33*, *rpl36*	11
	Small subunits of ribosome	*rps2*, *rps3*, *rps4*, *rps7* ^a^, *rps8*, *rps11*, *rps12* ^a^, *rps14*, *rps15*, *rps16*, *rps18*, *rps19*	14
	RNA polymerase	*rpoA*, *rpoB*, *rpoC1*, *rpoC2*	4
	Ribosomal RNA genes	*rrn4.5S* ^a^, *rrn5S* ^a^, *rrn16S* ^a^, *rrn23S* ^a^	8
	Transfer RNA genes	*trnA-UGC* ^a^, *trnC-GCA*, *trnD-GUC*, *trnE-UUC*, *trnF-GAA*, *trnfM-CAU*, *trnG-GCC*, *trnG-UCC*, *trnH-GUG*, *trnI-CAU* ^a^, *trnI-GAU* ^a^, *trnK-UUU*, *trnL-CAA* ^a^, *trnL-UAA*, *trnL-UAG*, *trnM-CAU*, *trnN-GUU* ^a^, *trnP-UGG*, *trnQ-UUG*, *trnR-ACG* ^a^, *trnR-UCU*, *trnS-GCU*, *trnS-GGA*, *trnS-UGA*, *trnT-GGU*, *trnT-UGU*, *trnV-GAC* ^a^, *trnV-UAC*, *trnW-CCA*, *trnY-GUA*	37
Other genes	Protease	*clpP*	1
	Maturase	*matK*	1
	Translation initiation factor	*infA*	1
	Envelop membrane protein	*cemA*	1
	Subunits of acety-CoA-carboxylase	*accD*	1
	C-type cytochrome synthesis	*ccsA*	1
Unknow gene	Hypothetical chloroplast reading frames	*ycf1*, *ycf2* ^a^, *ycf3*, *ycf4*	5
Total			130

Note: ^a^ indicates that it contains two copies of genes.

**Table 4:** SSR statistics of chloroplast genomes of *S. japonica* var. *timoriensis* and *S. japonica* var. *discolor*.

Types and Regions	Name	*S. japonica* var. *timoriensis*	*S. japonica* var. *discolor*
Types	mononucleotide	168	176
	dinucleotide	18	18
	trinucleotide	7	6
	tetranucleotide	9	7
	pentanucleotide	5	4
Regions	LSC	142	143
	SSC	45	48
	IR	20	20
Total		207	211

## Data Availability

The genome sequence data generated in this study are openly available in GenBank of NCBI at (https://www.ncbi.nlm.nih.gov/) under the accession no. PP175339-PP175340.
